# Clinicopathological Evaluation of Papillary Thyroid Microcarcinoma

**DOI:** 10.7759/cureus.56404

**Published:** 2024-03-18

**Authors:** Ando Takahito, Kimihito Fujii, Hirona Banno, Masayuki Saito, Yukie Ito, Mirai Ido, Manami Goto, Yukako Mouri, Junko Kousaka, Tsuneo Imai, Shogo Nakano

**Affiliations:** 1 Division of Breast and Endocrine Surgery, Department of Surgery, Aichi Medical University, Nagakute, JPN

**Keywords:** pten, extrathyroidal invasion, thyroid capsule invasion, lymph node metastasis, papillary thyroid microcarcinoma

## Abstract

Background and aims: Clinicians sometimes encounter papillary thyroid microcarcinoma (PMC) that is less than 10 mm, associated with lymph node metastasis. In this study, we assessed PMC clinicopathologically to clarify risk factors for poor prognosis.

Patients and methods: Fifty-one patients who underwent thyroid surgery at Aichi Medical University from September 2009 to October 2016 were included. Patients were divided into two groups, pEX-positive (23 patients) and pEX-negative (28 patients), based on the pathological finding of thyroid capsule invasion. The former indicates that the tumor infiltrated the thyroid capsule and spread to the neighboring tissue, and the latter indicates no capsule invasion. We analyzed factors such as patient characteristics, pathological findings, and serum levels of thyroid hormones in the two groups.

Results: No statistical differences were observed between the two groups in gender distribution or age at surgery. Preoperative cancer diagnoses were established for more patients in the pEX-positive group than in the pEX-negative group (n = 21 and 14, respectively; P = 0.004). The mean (±SD) pathological tumor diameter was 5.42 ± 2.77 in the pEX-negative group and 8.32 ± 1.61 in the pEX-positive group (P < 0.001). No significant differences in preoperative serum levels of free T3, free T4, thyroid-stimulating hormone, or thyroglobulin were observed between the two groups. The odds ratio for node positivity in tumors invading thyroid capsules (pEX-positive) compared to those with no capsule invasion (pEX-negative) was 13.20 (95% confidence interval, 3.45-50.42). Immunohistological staining for phosphatase and tensin homolog deleted from chromosome 10 (PTEN) and Akt (protein kinase B) revealed the facilitation of PTEN and suppression of Akt, which might indicate downregulation of the phosphoinositide 3-kinase-Akt (PI3K-Akt) cascade.

Discussion: In general, the prognosis of PMC is favorable. However, the prognosis is less favorable in patients with nodal metastasis or extrathyroidal invasion. It is controversial whether resection is required for proven PMCs. For PMCs associated with extrathyroidal invasion, regional lymph node resection with lobectomy should be performed due to the high risk for lymphatic spread. There might be a possibility that the natural progression of PMC seems to be controlled by the facilitation of PTEN. However, a tumor in the lateral peripheral region of the thyroid parenchyma might be associated with capsule invasion followed by lymphatic spread.

## Introduction

Papillary thyroid microcarcinomas (PMCs) smaller than 10 mm in diameter are being detected while they remain asymptomatic due to advances in ultrasonographic imaging and ultrasonographic-guided fine-needle aspiration biopsy. The probability of enlargement for PMCs is extremely slow. In one study, 93.3% of 346 PMCs did not grow by more than 3 mm during five years of follow-up [1]. Because some clinical and pathological findings strongly suggest that most PMCs can be considered harmless and non-life-threatening [1-4], observation without surgery by dedicated physicians, which is called active surveillance, could be an alternative therapeutic strategy. However, clinicians sometimes encounter PMCs with lymph node metastasis that require resection. In this study, we analyzed the clinicopathological findings of PMCs, including incidentally diagnosed cases in resected specimens with the diagnosis of benign thyroid disease. This article is the first one that analyzed the feature of PMCs with immunohistochemical staining technique and evaluated statistically the risk of lymph node metastasis.

A part of this article was previously presented as a meeting abstract at the 16th Biennial Congress of the Asian Association of Endocrine Surgeons on 8-10 March 2018 and the 38th ESSO Congress on 10-12 October 2018.

## Materials and methods

Patients

The medical records of 51 patients who underwent thyroid surgery from September 2009 to October 2016 with a pathological diagnosis of PMC and pathological diameter less than 10 mm were retrospectively assessed. All patients enrolled were treated at Aichi Medical University Hospital in Nagakute, Japan. We analyzed clinicopathological data, which included patient characteristics, preoperative laboratory data, and pathological findings of the resected specimens. Patients were divided into two groups, pEX-positive (23 patients) and pEX-negative (28 patients), based on the pathological finding of thyroid capsule invasion. The former indicates that the tumor infiltrated the thyroid capsule and spread to the neighboring tissue, and the latter indicates no capsule invasion. Patients’ characteristics are summarized in Table 1. All data were from the date of surgery. As shown in Table 1, benign tumors (n = 16) included adenomatous goiter (n = 12), follicular tumor (n = 3), and Basedow’s disease (n = 1).

**Table 1:** Patient characteristics * Diameter was measured with preoperative radiological imaging.

	pEX-negative	pEX-positive	P-value
Gender			
Male	4	4	0.761
Female	24	19	
Age, years (mean ± SD)	58.2 ± 13.3	52.8 ± 12.6	0.147
Presence of neck tumor			
Yes	16	7	0.056
No	12	16	
Tumor diameter*, mm (mean ± SD)	25.1 ± 23.0	11.2 ± 7.2	0.143
Preoperative diagnosis			
Benign tumor	14	2	0.001
Cancer	14	21	
Operative procedure			
Total thyroidectomy	13	13	0.473
Hemi-thyroidectomy	15	10	

Tumor samples

Pathological assessment of resected thyroid specimens was performed by Aichi Medical University Hospital’s Department of Pathology. Histopathological grading was based on the eighth edition of the tumor, node, and metastasis (TNM) classification system [5]. Briefly, papillary carcinomas are predominantly solid, although small cystic foci can be identified. Those carcinomas usually are infiltrative and their margins often are poorly defined. The diameter of PMCs enrolled in this study was measured microscopically by the pathologists.

Immunohistochemistry

Immunohistochemical staining was performed on the BenchMark XT automated staining system (Ventana Medical Systems, Oro Valley, AZ, USA). The following antibodies were used: phospho-Akt (1:25; Cell Signaling Technology, Tokyo, Japan), PTEN (1:100; Cell Signaling Technology, Tokyo, Japan), vascular endothelial growth factor (VEGF) (1:50; Santa Cruz Biotechnology, Dallas, TX, USA), E-cadherin (1:500; Cosmo Bio USA, Carlsbad, CA, USA), MMP-2 (1:50; Atlas Antibodies AB, Bromma, Sweden), CXCR4 (1:100; Miltenyi Biotec, Bergisch Gladbach, Germany), and p38 MAPK (1:500; Proteintech Group, Rosemont, IL, USA).

Immunohistochemistry assessment

Evaluation of immunostained slides was performed in random order by a single pathologist blinded to the other data of the paired samples. The slides were assessed with optical microscopy at 20x magnification. The staining intensity for each marker was graded as none or positive. Positive staining intensity ranged from light yellow to dark brown. The percentage of immunoreactivity was also graded as none or positive. Positive immunoreactivity was defined as >10% immunopositive cells distributed in the cancer nest. Generally, the immunoreactivity is graded into three or four steps [6]. However, the cancer nests of the patients enrolled in this study are so small that we could not investigate them closely. Therefore, we made over the grading of immunoreactivity as a simple system.

Statistical analysis

All data were analyzed using Bell Curve for Excel (Social Survey Research Information, Tokyo, Japan). The significance level was defined as 0.05. The variables such as age and thyroid-stimulating hormone (TSH) were assessed using Student’s t-test. The Mann-Whitney U test was performed for tumor diameter, free-T3, free-T4, and thyroglobulin concentration because those factors did not show normal probability distribution. Categorical variables were analyzed using the chi-square test. The odds ratio was calculated and compared for three parameters: symptomatic tumor, preoperative diagnosis, and nodal status. The cumulative survival rate of each group was estimated using the Kaplan-Meier method and compared using the log-rank test.

## Results

Patient characteristics

In the pEX-negative group, approximately 46% of patients (13/28) underwent thyroidectomy due to physical symptoms such as neck tumors or hoarseness. The tumor diameter in this group, which was evaluated radiologically, was relatively large. Median tumor diameter was 18 mm (range, 5-71 mm). Not surprisingly, half of the patients in the pEX-negative group had a preoperative diagnosis of benign tumor. In other words, half of the cancers were diagnosed incidentally based on pathological evaluation of the resected specimens postoperatively (Table 1).

Preoperative laboratory data

PMCs were small and did not produce hormones. There were no significant differences in any preoperative laboratory variables between the two groups. Thyroglobulin was not elevated above the upper limit of the reference range in patients with cancer (Table 2).

**Table 2:** Preoperative laboratory data Data are expressed as means ± SD.

	pEX-negative	pEX-positive	P-value
Free T3 (pg/ml)	2.89 ± 0.48	2.83 ± 0.28	0.608
Free T4 (pg/ml)	1.07 ± 0.15	1.03 ± 0.08	0.267
Thyroid-stimulating hormone (mIU/ml)	1.12 ± 0.90	1.53 ± 1.22	0.184
Thyroglobulin (ng/ml)	217.4 ± 839.0	186.4 ± 739.3	0.098

Prognosis

During the follow-up period, we recognized three recurrences. In one patient of the pEX-negative group, a locally recurrent tumor around the trachea was found. Two patients had lung metastases, one in each group. Three patients died; the cause of death was breast cancer in two patients and renal failure in one patient. Mean survival after surgery was 10.59 ± 0.47 (standard error, SE) years in the pEX-negative group and 8.25 ± 0.00 (SE) years in the pEX-positive group. The number of events was so small that the difference in survival rate between the two groups did not reach statistical significance (P = 0.201, log-rank test).

Pathological findings and odds ratio

As half of the pEX-negative group had incidentally diagnosed PMC, the mean pathological diameter of cancerous lesions in the pEX-negative group was significantly smaller than that of the pEX-positive group, 5.42 ± 2.77 versus 8.32 ± 1.61 cm (P = 0.0009) (Table 3).

**Table 3:** Summary of pathological findings

	pEX-negative	pEX-positive	P-value
Tumor diameter, mm (mean ± SD)	5.42 ± 2.77	8.32 ± 1.61	0.0009
Nodal metastasis			
Yes	6	18	<0.0001
No	22	5	
Multiple lesions			
Yes	6	8	0.287
No	22	15	

The most interesting finding was a significant difference in nodal metastasis status. Thyroid capsule invasion might have been the reason for lymphatic metastasis. There were five patients with nodal metastases in the pEX-negative group and 18 patients in the pEX-positive group (P < 0.0001) (Table 3). The odds ratio for nodal metastasis among tumors with thyroid capsule invasion (pEX-positive) compared to tumors with no capsule invasion (pEX-negative) was 13.20 (95% confidence interval, 3.45-50.42) (Table 4).

**Table 4:** Odds ratios of each parameter

Parameter		Odds ratio	95% CI
Symptomatic tumor	pEX-negative vs. pEX-positive	2.956	0.950-9.192
(Yes vs. no)			
Preoperative diagnosis	pEX-positive vs. pEX-negative	8.531	1.680-43.306
(Cancer vs. benign)			
Nodal metastasis	pEX-positive vs. pEX-negative	13.200	3.455-50.429
(Yes vs. no)			

Immunohistochemical staining

To assess the signaling pathway regulating tumor progression, we first focused on the phosphoinositide 3-kinase (PI3K)-protein kinase B (Akt) pathway, which controls a myriad of cellular processes and is implicated in cancer cell growth [6]. We used phospho-Akt antibodies because Akt regulates key cellular processes such as apoptosis, protein synthesis, metabolism, and cell cycle subsequent to the activation of PI3K [7]. However, none of the patients had overexpression of phospho-Akt (Table 5).

**Table 5:** Results of immunohistochemical staining using seven types of antibodies MAPK: mitogen-activated protein kinase; PTEN: phosphatase and tensin homolog deleted from chromosome 10; VEGF: vascular endothelial growth factor; CXCR4: CXC chemokine receptor type 4; MMP-2: matrix metalloproteinase 2; NA: not assessed.

Antibody and expression (location)	pEX-negative	pEX-positive	P-value
Phospho-Akt (cytosol)			
Positive	0	0	0.175
Negative	22	22	
NA	6	1	
p38 MAPK (cytosol)			
Positive	21	22	0.125
Negative	1	0	
NA	6	1	
PTEN (cytosol)			
Positive	22	22	0.175
Negative	0	0	
NA	6	1	
VEGF (cell membrane)			
Positive	10	13	0.214
Negative	12	8	
NA	6	1	
CXCR4 (cell membrane)			
Positive	9	13	0.073
Negative	12	9	
NA	7	1	
MMP-2 (cell membrane)			
Positive	21	22	0.125
Negative	1	0	
NA	6	1	
E-cadherin (cell membrane)			
Positive	21	22	0.125
Negative	1	0	
NA	6	1	

The opposite function is exerted by a lipid phosphatase called phosphatase and tensin homolog deleted from chromosome 10 (PTEN), which is a major suppressor that breaks down the PI3K-Akt pathway [8]. All patients had a positive reaction to anti-PTEN antibodies. Based on these results, the PI3K-Akt pathway seemed to be suppressed by PTEN activation. Next, the mitogen-activated protein kinase (MAPK) pathway, which is another signaling pathway that regulates a wide variety of essential cellular processes, including proliferation, differentiation, apoptosis, and stress responses, is considered a main driving force for the proliferation of papillary thyroid carcinoma if mutations result in the activation of this pathway [9,10]. We focused on the activated p38 MAPK pathway, which is frequently a treatment target [11-13]. Thyroid carcinoma is predominantly a MAPK-driven cancer and about 70% of them are caused by mutations that facilitate this pathway [14].

**Figure 1:**
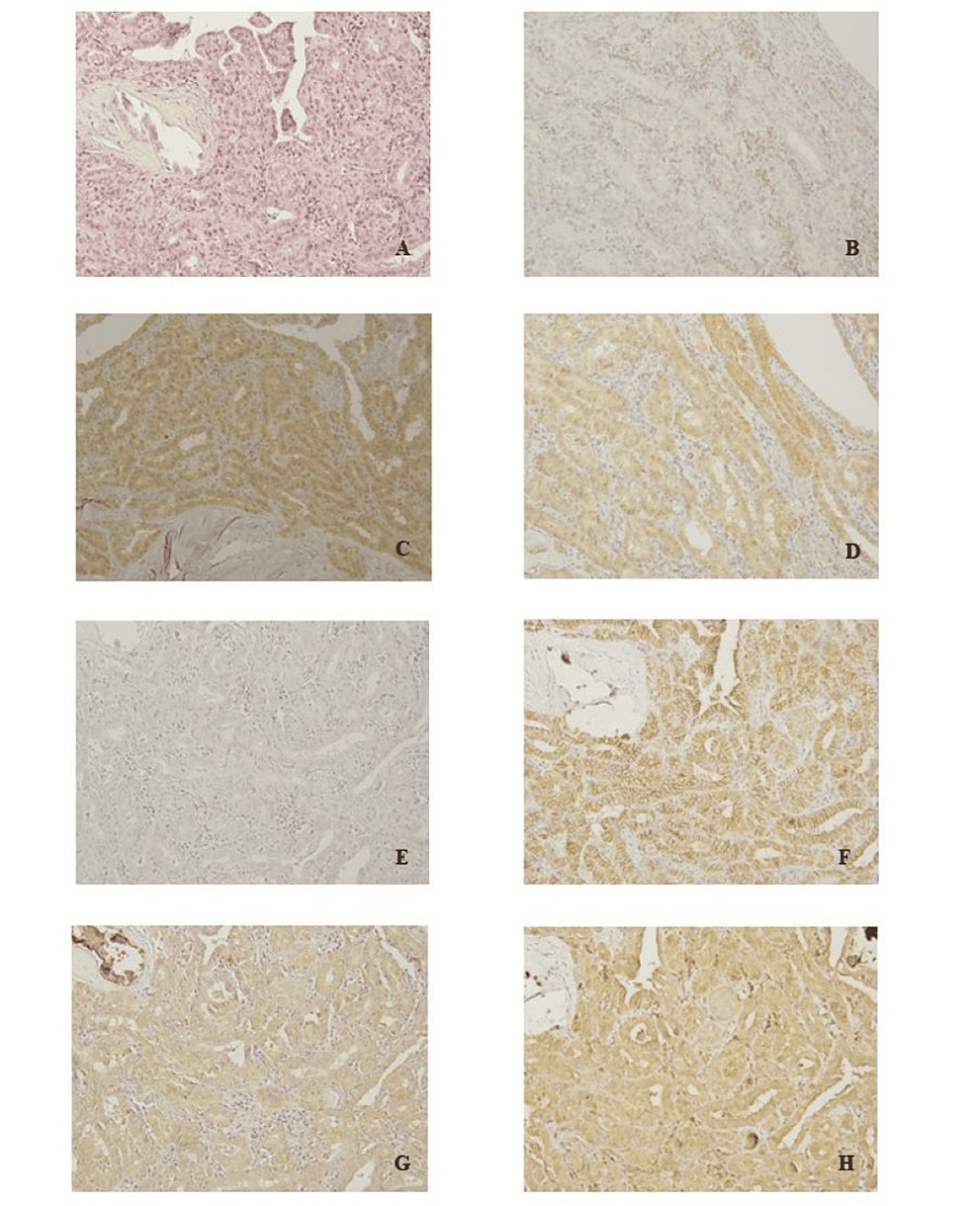
Microscopic findings Microscopic findings in a patient who was in the pEX-positive group. Hematoxylin–eosin (HE) and immunohistochemical staining results are presented in semi-serial sections. The list includes the name of the antibody and the reaction. Magnification, ×20. The assessments of the staining are listed as follows: (A) HE; (B) phospho-Akt, negative; (C) PTEN, positive; (D) CXCR4, positive; (E) VEGF, negative; (F) E-cadherin, positive; (G) MMP-2, positive; (H) p38 MAPK, positive. Those staining results can be referred to in Table 6. HE: hematoxylin and eosin; MAPK: mitogen-activated protein kinase; PTEN: phosphatase and tensin homolog deleted from chromosome 10; VEGF: vascular endothelial growth factor; CXCR4: CXC chemokine receptor type 4; MMP-2: matrix metalloproteinase 2.

As expected, all patients in the pEX-negative group except one had a positive reaction to anti-p38 MAPK antibodies. Regardless of small tumor size, the p38 MAPK pathway was confirmed to be upregulated in thyroid papillary carcinoma. CXC chemokine receptor type 4 (CXCR4), which induces lymphatic proliferation, was evaluated in the immunostaining study. Liang et al. reported strong expression in patients with metastatic thyroid papillary carcinoma [15]. However, the two groups had similar reactions in our study. VEGF, an angiogenic factor, was reported to be associated with tumorigenesis and metastasis in several cancers [13,15]. The expression of VEGF was similar in the two groups. Matrix metalloproteinase (MMP)-2 is considered to contribute to cancer progression and metastasis by degrading type IV collagen, the most abundant component of the basement membrane [16,17]. In our study, almost all of the patients had strong MMP-2 expression. E-cadherin is expressed on the surface of cells and creates cell-cell adhesion. The loss of E-cadherin might disrupt cellular adhesion and lead to cancer metastasis [18]. It has been reported that the downregulation of E-cadherin will promote epithelial-mesenchymal transition in thyroid cancer [19]. Almost all patients had strong reactions to E-cadherin; no significant differences between the two groups were observed. It is controversial whether E-cadherin immunoreactivity reflects the behavior of thyroid papillary carcinoma [20]. However, the expression of E-cadherin in recurred or metastasized thyroid carcinomas was low or lacking [21]. The strong reactions of E-cadherin in our patients may suggest that PMCs have a low likelihood of metastatic spread. The relationships between E-cadherin expression and lymph node metastases could not be clarified, unfortunately. The results of immunohistochemical assessments are summarized in Table 5. Figure 1 demonstrates microscopic findings of example. Table 6 indicates the results of immunohistochemical staining expression.

**Table 6:** The results of the immunohistochemical staining expression This table explains the staining results of seven types of antibodies that are presented in Figure 1. HE: hematoxylin and eosin; MAPK: mitogen-activated protein kinase; PTEN: phosphatase and tensin homolog deleted from chromosome 10; VEGF: vascular endothelial growth factor; CXCR4: CXC chemokine receptor type 4; MMP-2: matrix metalloproteinase 2.

A: HE	B: Phospho-Akt, negative
C: PTEN, positive	D: CXCR4, positive
E: VEGF, negative	F: E-cadherin, positive
G: MMP-2, positive	H: p38 MAPK, positive

## Discussion

Recently, whether operative treatment is indicated for PMC is frequently discussed. Since PMCs are slowly growing, active surveillance, which means continued observation without immediate surgery, can be appropriate [2]. In a report on 1,235 patients with PMC who were followed for 75 months on average, Ito et al. demonstrated that only 4.6% of patients had tumor enlargement [4]. Mortality related to PMC is low; no deaths due to PMC progression were recognized in a large clinical trial with 1,235 patients with PMC conducted by Miyauchi et al. [22,23]. However, clinicians sometimes encounter PMCs with lymph node metastasis, which means that the patient would be diagnosed with advanced thyroid cancer. Thus, we conducted this retrospective study to evaluate the biological features of PMC using statistical analysis and immunohistochemical staining.

Dividing the enrolled patients into two groups according to the pathological finding of thyroid capsule invasion, pEX-negative and pEX-positive, the latter group included a significantly larger number of patients with lymph node metastasis than the former group. Capsule invasion might induce metastatic spread. To clarify biological differences between the two groups, we performed immunostaining examinations of the tumors. First, the expression of CXCR4, which induces lymphatic spread, was similar in the two groups. VEGF, which is a potent inducer of angiogenesis around cancer, showed no significant trend toward overexpression in the pEX-positive group. However, the overexpression of MMP-2, which releases bioactive VEGF, was observed in almost all patients. However, it is difficult to explain this phenomenon because MMP-2 did not facilitate VEGF expression in the study patients [24]. A trend toward VEGF overexpression was expected but not observed in the pEX-positive group. We have one possible hypothesis for this discrepancy.

As a driving force of tumor progression, the PI3K-Akt pathway is considered to be essential. In thyroid papillary carcinoma, somatic mutations in *PIK3CA* and *AKT1* are found in limited cases [25]. It was also recognized in our immunostaining study using anti-phospho-Akt antibodies. No patients with overexpression were found in our series. PTEN, a tumor suppressor, is a major brake of the PI3K-Akt pathway [8]. Our immunostaining analyses revealed the facilitation of PTEN. Although this phenomenon was observed only by the immunostaining analyses, a hypothesis that the PI3K-Akt pathway was suppressed by the upregulation of PTEN might be proposed. This inverse expression of PTEN and Akt downregulates the PI3K-Akt pathway and suppresses the expression of both CXCR4 and VEGF. Expression of CXCR4 and VEGF is modulated by the activity of the PI3K-Akt pathway [25,26].

We planned to clarify the mechanisms of how pEX-positive tumors have the potential for lymphatic spread. The immunohistochemical evaluation of microvascular invasion did not clarify the mechanism. The fact that the cancerous lesion is located in the peripheral region of the thyroid parenchyma might be a notable factor for thyroid capsule invasion followed by lymphatic spread. Ito et al. mentioned that clinicians should pay attention to tumors located in the peripheral region. They also mentioned that tumors located on the course of the recurrent laryngeal nerve or attaching the trachea at an obtuse angle were also candidates for surgery [2]. The capsules of thyroid grand consisted of two layers, such as true and false capsules. The thin fibro-elastic capsule called a true capsule surrounds the thyroid gland. This capsule, in turn, is covered by pre-tracheal fascia, which is considered a false capsule. The true capsule gives rise to septa deep into the thyroid parenchyma dividing the gland into the lobules. The septa give passage for the blood vessels, nerves, and lymphatics into the gland [27]. If the cancer located in the peripheral region invades the true capsule and the surrounding organs, lymphatic spread might occur through those micro-vessels in the true capsule.

This study has several limitations. First, the number of study patients was limited. Second, immunostaining analyses could not be performed on every cancer specimen because some tumors were so small that we could not make a sufficient number of thin slice sections containing the cancerous lesions. Immunostaining analyses can only produce a qualitative assessment, therefore a quantitative assessment, such as western blotting, might be required to describe the real biological features of PMC.

## Conclusions

As one of the natural features of PMC, the downregulation of the PI3K-Akt pathway was recognized. An interesting feature of PMC is that capsule invasion by the cancerous lesion might induce lymph node spread, although the biological etiology for activation of this phenomenon was not confirmed in this study. Location in the lateral peripheral region of the thyroid parenchyma might play a role in capsule invasion followed by lymphatic spread.
